# Determinants of pressure to conceive among reproductive age women in Sub-Saharan Africa: A multilevel analysis of recent Demographic and Health Surveys in five countries

**DOI:** 10.1371/journal.pgph.0004244

**Published:** 2025-02-10

**Authors:** Alemneh Tadesse Kassie, Alebachew Ferede Zegeye, Astewil Moges Bazezew, Ephrata Yetayeh Mamo, Demiss Mulatu Gebru, Tadesse Tarik Tamir

**Affiliations:** 1 Department of Clinical Midwifery, School of Midwifery, College of Medicine and Health Sciences, University of Gondar, Gondar, Ethiopia; 2 Department of Medical Nursing, School of Nursing, College of Medicine and Health Sciences, University of Gondar, Gondar, Ethiopia; 3 Department of Surgical Nursing, School of Nursing, College of Medicine and Health Sciences, University of Gondar, Gondar, Ethiopia; 4 Institute of public health, College of Medicine and Health Sciences, University of Gondar, Gondar, Ethiopia; 5 Department of Health System and Policy Institute of Public Health, College of Medicine and Health Sciences, University of Gondar, Gondar, Ethiopia; 6 Department of Pediatrics and Child Health Nursing, School of Nursing, College of Medicine and Health Sciences, University of Gondar, Gondar, Ethiopia; University of Ghana, GHANA

## Abstract

Globally, 40% of pregnancies are unplanned, with higher rates in Sub-Saharan Africa, often ending in abortion. Women face pressure from spouses/families to conceive, leading to unintended pregnancies and violations of reproductive rights, jeopardizing women’s autonomy and well-being. Respecting individual decisions is crucial during this pivotal life stage. The study conducted a secondary data analysis using information from the latest Demographic and Health Surveys, encompassing five Sub-Saharan African nations from 2021 to 2023. The research focused on a weighted sample of 97,350 married women of reproductive age. Data analysis was performed using Stata 14, employing a multilevel mixed-effects logistic regression model to uncover the factors contributing to the pressure exerted by husbands and families on women to conceive. The study highlights that in Sub-Saharan Africa, approximately one in ten women experience pressure to conceive. Factors like age (20-35 years; AOR = 1.6, 95% CI: 1.46, 1.87), socioeconomic status (middle/affluent; AOR = 1.12, 95% CI: 1.04, 1.2 and AOR = 1.12, 95% CI: 1.03, 1.21), parity (childless; AOR = 4.65, 95% CI: 4.1, 5.2), and community literacy (low; AOR = 1.44, 95% CI: 1.25, 1.66) significantly influence this pressure. Notably, women in Tanzania have a 55% lower risk (AOR = 0.45, 95% CI: 0.41, 0.49), while those in Mozambique face a 1.88 times higher risk. The study highlights the substantial pressure faced by reproductive-age women to conceive in Sub-Saharan Africa. Addressing these challenges through targeted policies and interventions is crucial to empower women and promote their reproductive autonomy.

## Introduction

An unplanned pregnancy is one that occurs as a result of mistimed or unwanted conception. Worldwide, unplanned pregnancies account for about 40% of all pregnancies, with half of them ending in abortion. One possible explanation for this occurrence is that women may experience pressure to conceive from close partners or relatives [1]. Women may face pressure from their families and spouses to become pregnant unintentionally, and abusive partnerships can result in a host of issues, such as women often encounter two crises, unintended pregnancies and mental maltreatment. It is possible for her to seem as though she is losing authority or control [2,3]. Families and partners must remember that each woman has a unique experience, and that it is critical to respect individual decisions and well-being throughout this essential stage of life as motherhood.

In poor nations, pregnancy- and childbirth-related factors claim the lives of an estimated 70,000 teenage women annually. In underdeveloped nations, the primary causes of death for teenage girls who are older are childbirth and pregnancy [1]. Unplanned pregnancies are influenced by the pressures of family and partners [4]. Unplanned pregnancies are a serious health problem that raise the rate of maternal death [5]. It has been reported that women who become pregnant unexpectedly tend to have worse relationships with their spouses and are more vulnerable to emotional abuse than women who become pregnant on plan. Unplanned pregnancies have been linked to an increased prevalence of maternal depression symptoms [6–8]. Women’s health suffers from difficulties, particularly postpartum depression, and they may attempt to harm the baby after birth or have a stillbirth. Feelings of shame or regret may deter women from seeking timely medical care during pregnancy. Women with unintended pregnancies had 2.5 times the risk of experiencing depression during pregnancy and postpartum period compared to those with planned pregnancies [7,9].

The quality of life of a pregnant woman is influenced by various aspects, including her social environment, prior pregnancy experience, level of education, and economic status [10,11]. Pregnancy readiness and general quality of life are closely related to this situation for the women. Women who are expecting have both positive and negative effects from pregnancy-related changes. Pregnancy can be a source of joy, satisfaction, maturity, self-expression, and happiness as well as anxiety, anxious waiting, and burnout [12]. Unwanted pregnancies are those that happen when no children are intended, whereas mistimed pregnancies occur earlier than expected [13]. There were 121 million unintended pregnancies between 2015 and 2019, demonstrating that this is still a major impact and a global public health concern [14]. Unwanted pregnancy is a serious health risk and it is linked to increased rates of maternal and neonatal mortality and morbidity [15]. Unintended pregnancy was linked to lower birth weight, an increased risk of preterm birth (OR 1.15), and a higher likelihood of preeclampsia (OR 1.21) and the odds of requiring a cesarean section were 1.32 times greater [16]. Each year, approximately 42 million women worldwide with unintended pregnancies opt for abortion, with nearly half (20 million) being unsafe procedures. Unsafe abortions result in about 68,000 deaths annually [17]. Pressure from family, friends, and cultural expectations can make women feel obliged to have children. There is still a stigma attached to having a baby “too young” or “too late” in life [18–20]. In Sub-Saharan Africa (SSA), social norms often expect women to become mothers soon after marriage, typically within a few years, which can lead to unwanted pregnancies. Factors such as poor awareness, limited access to contraception, incorrect contraceptive use, supply irregularities, limited method choices, misinformation about side effects, and pressure from spouses and families all contribute to this issue [21].

Women who become pregnant owing to pressure may not pay attention to their health during or after birth [22]. While global fertility rates are decreasing, certain regions in Africa, such as Ghana, maintain relatively high fertility rates, with a total fertility rate of 3.9. In Ghana, women face significant societal pressure to bear children, which influences their reproductive choices and health outcomes. [23,24]. The emotional impact of pregnancy can put a strain on relationships. Navigating these challenges requires open communication between partners to ensure mutual understanding, support, and collaboration [25,26].

Decisions about parenthood should be voluntary and mutual, based on respect, empathy, and understanding. In SSA, societal norms often pressure individuals to have children, with parenthood seen as the default life path [27]. Reproductive coercion occurs when some partners pressure women into becoming pregnant or continuing a pregnancy against their wishes through tactics like verbal threats, coercion, or interference with contraception. This behavior is both unacceptable and harmful [28].

Child-rearing in Africa is challenging due to age, health, and economic conditions. Raising a child can be labor-intensive, costly, and time-consuming, especially when women are not adequately prepared. This is also the case in wealthy nations, where many workplaces are not structured to effectively support new mothers with children [29]. Moms often cut back on their paid job hours after having a baby in order to take on additional childcare duties [30]. In Africa, challenges like war, poverty, migration, and hunger are linked to uncontrolled population growth. Limited contraceptive use allows unrestricted childbirth, impacting women’s health and economic well-being. [31–33]. We may not support China’s government’s recommendation to limit the number of children per woman or parent, as it violates reproductive rights [34]. However, it is important to empower women to make their own decisions and educate them to deal with pressure. According to studies, men in abusive relationships may employ techniques to force women to become pregnant, such as destroying their birth control [35].

The Sustainable Developmental Goal (SDG) agenda includes metrics aimed at preventing teenage pregnancy and childbirth in particular [36]. However, social stigma and rumors may also be connected to gossips regarding infertility may begin to spread among neighbors when they find out that a woman has not been pregnant after a certain period of time. According to research, one out of every four Australian women will experience spousal abuse at some point in her life [37]. Family violence can take various forms, including controlling behaviors such as banning women from friends and family, as well as physical, emotional, and sexual abuse [38].

When a woman gives birth to multiple children of the same gender, either as girls or boys, her spouse and family need a child of a different gender. We believe that women should be allowed to make their own decisions and live lives free from violence. It is estimated that 644,00 births to 15–19-year-olds occurred in Sub-Saharan Africa (SSA) in 2021, compared to only 68,00 births recorded in Central Asia. Comparatively, 332,000 teenagers in SSA (Sab-Saharan Africa) were between the ages of 10 and 14; in contrast, in South-East Asia was only 22,000 [39]. Efforts in SSA focus on preventing unplanned pregnancies, combating child marriage, promoting planned conception, and providing comprehensive support for women’s physical, mental, and financial readiness for childbirth.

## Methods

The five nations included in the Demographic and Health Surveys (DHS) in 2019 had the latest data available. To enable multilevel analysis, the different data sets were chosen; the datasets from the 2021 DHS across five nations were selected for their recent and comprehensive maternal health data. These nations represent diverse demographic and socio-economic conditions, providing key variables related to women’s reproductive health, including the pressure to become pregnant. This selection enabled a multilevel analysis, incorporating both individual-level factors and broader context-specific influences on reproductive decision-making. By accounting for personal, societal, and policy-related pressures, the analysis effectively addressed the complexities of maternal mortality and unintended pregnancies, making these datasets particularly relevant for our research focus.

The DHS data sets come from surveys that are nationally representative and include at least one woman who is of reproductive age, often between the ages of 15 and 49. A subsample of women of reproductive age was used for the secondary analysis of the most recent demographic and health survey information. The study employed Stata 14 statistical software for analysis, and a multilevel logistic regression model was utilized to identify weight determinants and associated factors of prevalence. The adjusted odds ratio’s P-value of less than 0.05 was utilized to determine which factors were substantially related to the result [40].

This study’s outcome variable was women’s pressure to become pregnant. pressure from a partner, their family, or the spouse’s relatives at any point throughout their marriage, including being told not to take contraception or to stop using it without a plan.

### Ethics-statement

This study was evaluated and found to have no ethical concerns after consultation with scholars experienced in human research ethics. The data utilized in this study was neither sensitive nor restricted. We accessed this data with proper consent. Additionally, because the data is publicly available, no further ethical approval was required.

### Study design and participants

This study employs a cross-sectional design to examine the pressure to conceive among women in SSA. The unit of analysis for this study is women who are in union (married or living together with a partner) and within the reproductive age range of 15 to 49 years. The study population consists of women aged 15 to 49 years who are in union, drawn from the most recent DHS datasets from multiple countries in Sub-Saharan Africa. This demographic was chosen to provide a comprehensive understanding of the factors influencing reproductive pressure within this specific age group and marital status.

### Study plan and period

A multilevel, community-based, cross-sectional study with mixed effects was carried out. A multilevel mixed effect analysis was performed using data from five SSA nations that were surveyed by the DHS between 2021 and 2023. As a component of the global DHS, the national DHS is carried out every five years utilizing pretested, validated, and structured instruments. Five years of DHS data (beginning in 2021) were collected in order to obtain a representative sample of recent DHS data from every region of SSA countries. The surveys have huge sample sizes, are population-based, and nationally representative of every nation.

### Study location

The region of Africa south of the Sahara is known as the sub-Saharan, and it is made up of four enormous and diverse regions: Eastern Africa, Central Africa, Western Africa, and Southern Africa. They have a combined population of 1.3 billion people living in an area of 9.4 million square miles. Based on data from the most recent DHS surveys, this study was carried out in five sub-Saharan African nations: Burkina Faso, Ghana, Kenya, Mozambique, and Tanzania.

### Population and requirements for eligibility

For this research, we employ the population and eligibility criteria. The initial demographic consisted of women residing in SSA nations. All women of reproductive age who fell into the designated enumeration zones included in the analysis made up the study population.

### Data source and sampling process

We appended the DHS survey data from five SSA countries together to examine the prevalence of pregnancy pressure among women of reproductive age. Every country’s survey has a varied set of statistics, some of which include information on fundamental health metrics including mortality, illness, fertility, and violence related to reproductive health, like pressure to become pregnant. Using a stratified two-stage cluster design, the DHS first creates the enumeration regions and then creates a sample of homes from each enumeration area in the second stage [41]. The dependent and independent variables for each country were extracted for this analysis using the individual record dataset, and the data was then appended using STATA.

The outcome variable (pressure to become pregnant) was created by recoding the variable “women pressure to become pregnant v636” from the individual record (IR) data set. To identify the variables linked to pressure to get pregnant, a binary logistic regression model was employed. The factors that influence the desire to become pregnant were expressed as an adjusted odds ratio with a 95% significance level. A P-value of less than 0.25 in the univariate analysis indicated that the data would be a good fit for the multivariable analysis. In multivariable logistic regression, all variables with P-value s <0.05 were considered statistically significant. The study included a weighted sample of 97,350 women in total “Table 1.”

**Table 1:** Sample size for individual and community-level determinants of women pressured to become pregnant among reproductive age group women in the SSA countries, DHS 2021–2023.

Country	Year of survey	Weighted sample (n)	Weighted sample (%)
Burkina Faso	2021	25,736	26.44
Ghana	2022	17,622	18.10
Kenya	2022	19,300	19.83
Mozambique	2022/23	16,390	16.84
Tanzania	2022	18,302	18.80
Total Weighted sample size		97,350	100.00

### Study variables

Dependent variables: The study’s outcomes were the frequency of women who experience pressure to become pregnant and the factors that affect women in the reproductive age range. The pressurized women were evaluated by recoding the variables V636 from the women’s status (WS) data set.

### Independent variables

Independent variables from two sources (variables at the individual and community levels) were taken into account for this analysis, given that DHS data are hierarchical. The independent factors at the individual level were: Women age (15–19, 20–35, 36–49), women education (have no formal education, primary, secondary, and higher), partner education (have no formal education, primary, secondary, and higher), Women earn as compared to husbands (less than, about the same, more than), husband occupation (have no occupation, have occupation), women have children (no, less than 5, more than 5), wealth index (poor, middle, rich), women media exposure (have no exposure, have exposure), husband age (under 20, 21–30, 31–39, >40), gender of household head (male, sex), and women’s contraceptive utilization (user, nonuser). The community-level independent variables were women’s residence (urban or rural), community-level media exposure (low or high), community-level illiteracy (low or high), and community-level poverty (low or high).

### Data entry and statistical evaluation

The data were extracted from recent DHS data sets and cleaned, recorded, and analyzed with STATA version 14 statistical software. The data were weighted using sampling weight, primary sampling unit, and strata before any statistical analysis to restore the representativeness of the survey and take into account the sampling design when calculating standard errors to get reliable statistical estimates. We used the weighting variable (v636) as a relative weight normalized to make the analysis survey-specific, while for the pooled data, we de-normalized the women’s pressured to become pregnant individual standard weight variable by dividing the pressured women individual standard weight by the sampling fraction of each country: (women pressured to become pregnant=V636× (total pressured to become pregnant women aged 15–49 years in the country at the time of the survey)/ (number of pressured to become pregnant women aged 15–49 years in the survey).

The assumptions of the standard logistic regression model, such as independence of observations and equal variance, are broken due to the hierarchical nature of the DHS data. For instance, women are nested within a cluster, and we assume that study subjects in the same cluster may share similar characteristics with participants in another cluster, which violates the independence observations and equal variance assumptions between clusters of the ordinal logistic regression model. This suggests that using a sophisticated model to take into account between-cluster factors is necessary. Given this, multilevel mixed-effects logistic regression was used to determine the factors associated with women being pressured to become pregnant. Multilevel mixed effect logistic regression follows four models: the null model (outcome variable only), mode I (only individual level variables), model II (only community level variables), and model III (both individual and community level variables). The model without independent variables (the null model) was used to check the variability of women pressured to become pregnant prevalence rates across the cluster. The association of individual-level variables with the outcome variable (Model I) and the association of community-level variables with the outcome variable (Model II) were assessed. In the final model (Model III), the association of both individual and community-level variables was fitted simultaneously with the outcome variable.

### Random effects (Measures of variation)

The prevalence rates of women under pressure to become pregnant varied throughout clusters, and random effects or measures of variation, such as the Likelihood Ratio Test (LR), Intra-class Correlation Coefficient (ICC), and Median Odds Ratio (MOR), were calculated to measure this variance. The ICC uses clusters as a random variable to quantify the degree of heterogeneity in prevalence rates of women under pressure to become pregnant between clusters. This ratio, which represents the portion of the total observed variation in women under pressure to become pregnant that can be associated with variations between clusters, is calculated as ICC = The median value of the odds ratio between the cluster at high likelihood of women being pressured to become pregnant and the cluster at lower risk when individuals are randomly selected from two clusters is known as the Median Odds Ratio (MOR), which quantifies the variation or heterogeneity in women being pressured to become pregnant prevalence rates between clusters in terms of the odds ratio scale; MOR = *e*0.95√VC.^C^ Moreover, the PCV demonstrates the variation in the women pressured to become pregnant prevalence rates explained by determinants and computed a PCV; where V-null = variance of the null model and VC = cluster level variance [42,43]. The probability of women being pressured towards becoming pregnant was estimated using fixed effects about independent factors at the individual and community levels. With a P-value of less than 0.05, the adjusted odds ratio and 95% confidence intervals were used to evaluate it and show its strength. Deviance = -2 (log-likelihood ratio) was used to compare the models due to the nested nature of the model; the model with the lowest deviance and the highest log likelihood ratio was chosen as the best-fit model.

We estimated the relationship between prevalence rates of women being urged to get pregnant and independent variables at the individual and community levels using fixed effects. Using an adjusted odds ratio and 95% confidence intervals with a P-value of less than 0.05, it was evaluated and its strength was displayed. Due to the hierarchical structure of the model, models were compared using deviance = -2 (log-likelihood ratio), and the best-fit model was determined by taking the lowest deviance and the highest log-likelihood ratio. By calculating the variance inflation factors (VIF), the variables employed in the models were checked for multi-collinearity; the results were within acceptable ranges of one to ten.

## Results

### Socio demographic and economic characteristics of study participants

A total of 97,350 women were included in the analysis of this study. Nearly two-thirds of participants were between the ages of 20 and 35, while nearly half of the participants’ husbands were over the age of 40. Regarding the educational status of the participants and their partners, more than one-third of them had no formal education. Around 40% of participants’ wealth index indicates that they are either rich or poor. Around three-quarters of study participants have less than five children at home. More than two-thirds of participants had experience with media exposure. Only 17 percent of women were the head of the household; the majority of participants’ spouses were employed; two-thirds of women enrolled in this study had about the same earnings as compared to their husbands (66.03%). Nearly two-thirds of women were non-users of any contraceptive method, and about two-thirds (68.7%) were residents of rural areas of Sub-Saharan Africa. “Table 2.”

**Table 2:** Socio-demographic and economic characteristics of study participants.

Individual level variables	Category	Frequency (n)	Percent (%)
Women age in years	15–19	5,878	6.04
	20–35	56,802	58.35
	36–49	34,670	35.61
Wealth index	Poor	38,744	39.80
	Middle	19,548	20.08
	Rich	39,058	40.12
Women education	No formal education	35,600	36.57
	Primary	31,140	31.99
	Secondary	25,292	25.98
	Higher	5,318	5.46
Husband/Partner education	No formal education	34,844	35.87
	Primary	28,518	29.36
	Secondary	25,566	26.32
	Higher	8,212	8.45
Women number of baby	Have no chilled	6,320	6.49
	Have less than 5 baby	72,934	74.92
	Have more than or 5 baby	18,096	18.59
women media exposure	No	28,656	29.44
	Yes	68,694	70.56
Husband or partners age	Under 20	1,042	1.07
	21–30	22,590	23.20
	31–39	27,464	28.21
	Above 40	46,254	47.51
Gender of house hold head	Male	80,382	82.57
	Female	16,968	17.43
Partner/husband employment	Non-employed	13,370	13.73
	Employed	83,980	86.27
Women earns compared to husband/partner	Less than	29,562	30.37
	About the same	64,282	66.03
	More than	3,506	3.60
Women utilization of contraception	User	36,974	37.98
	Non-user	60,350	62.02
Community level variables			
Household residence	Urban	33,880	34.80
	Rural	63,470	65.20
Community level of media exposure	Low	46,206	51.68
	High	43,204	48.32
Community level of illiteracy	Low	18,566	19.07
	High	78,784	80.93
Community level of poverty	Low	48,042	49.35
	High	49,308	50.65

### Prevalence of women being pressured to get pregnant by their family and spouses. (in five Sub-Saharan African countries)

**Fig 1:**
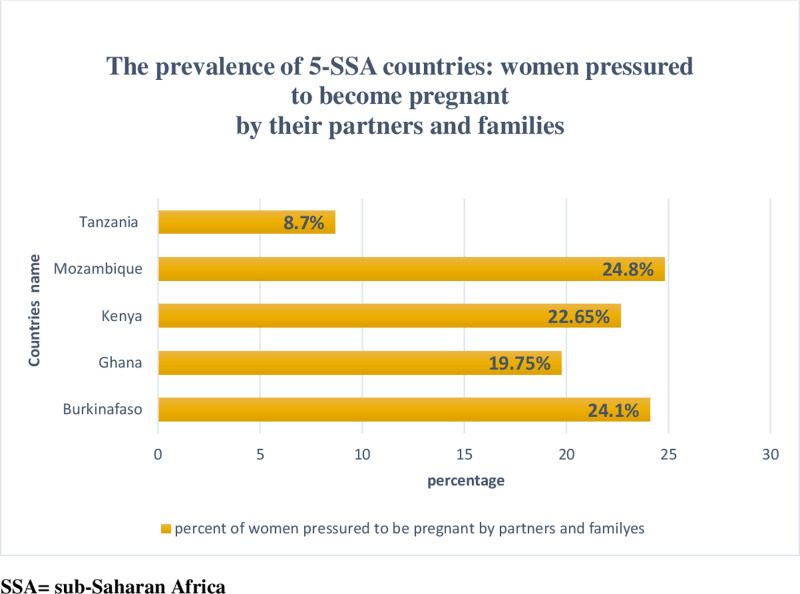
Prevalence of women being pressured to get pregnant by their spouses and family in five Sub-Saharan African (SSA) countries.

The frequency with which partners and family pressurize women to get pregnant varies greatly between SSA nations. As a result, Tanzania had the lowest rate of women experiencing pressure from their partners and families to become pregnant (8.7%), while Mozambique had the highest rate (24.8%) (Fig 1).

**Fig 2:**
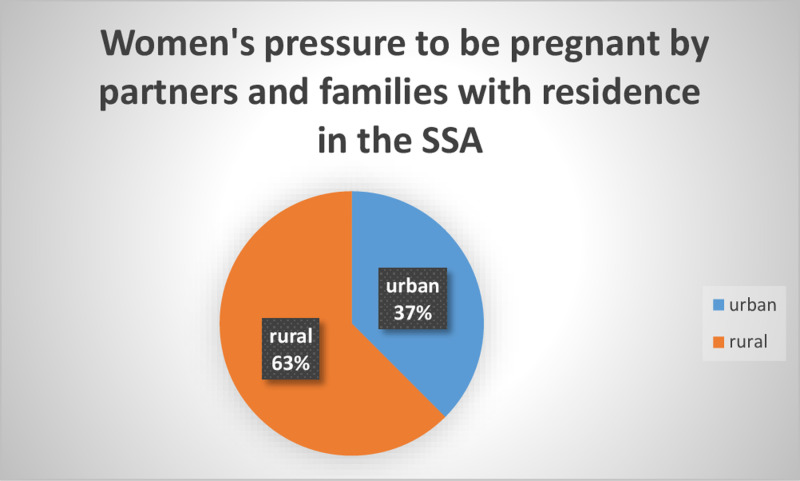
Pressure to become pregnant by spouses and families in SSA women, based on residence.

In SSA, the total prevalence of women’s partners and family pressuring them to get pregnant was 8.92% at a 95% confidence interval (8.74, 9.1). Compared to 37% of women in urban areas, 63% of women in rural regions reported feeling pressured to get pregnant by their partners and relatives (Fig 2).

### Random effect (Measures of variation) and model fitness

The findings from the null model and subsequent models (I, II, and III) provide a nuanced understanding of the factors contributing to the variation in women being pressured to become pregnant across communities. Initially, the null model revealed significant differences in the prevalence of women being pressured to become pregnant between communities, with a P-value of 0.000 and a variance of 0.5523755.

The intra-class correlation (ICC) value from model I indicated that approximately 14.8% of the variation in women being pressured to become pregnant can be attributed to disparities between communities. This suggests that community-level factors play a role in shaping the experiences of women in this regard. When community-level variables were included in the null model (model II), the ICC value decreased to 9.85%, indicating that the inclusion of community-level variables reduced the proportion of variation attributed to community-level differences. This suggests that individual-level factors within each community play a more significant role in shaping the experiences of women in this context.

In the final model (model III), which accounted for both individual and community-level variables, the ICC value was not reported. However, the likelihood of women being pressured to become pregnant varied by a factor of 1.78 times across low and high-pressure clusters. This indicates that the inclusion of both individual and community-level variables in the model resulted in a more nuanced understanding of the factors contributing to the variation in women being pressured to become pregnant.

The findings from these models suggest that both individual and community-level factors contribute to the variation in women being pressured to become pregnant. While community-level factors play a role, individual-level factors within each community are more significant in shaping the experiences of women in this regard. Future research should aim to identify specific individual and community-level factors that contribute to the pressure on women to become pregnant and to develop targeted interventions that address these factors “Table 3.”

**Table 3:** Model comparison and random effect analysis for partner and family pressures among women in the Sub-Saharan Africa countries.

Parameter	Null model	Model I	Model II	Model III
variance	0.5523755	0.4715545	0.3595246	0.3675845
ICC	14.37%	14.80%	9.85%	10.05%
MOR	2.026	2.051	1.770	1.780
PCV	Reference	14.63%	34.96%	33.45%
Model fitness				
LLR	-28626.323	-28046.525	-25276.091	-24719.463
Deviance	57,252.646	56,093.05	50,552.182	49438.926

ICC: intra-cluster correlation, LLR: log-likelihood ratio, MOR: median odds ratio, PCV: proportional change in variance.

Factors Influencing the Prevalence of Women Pressured to Become Pregnant in Sub-Saharan Africa:

In the final fitted model of multivariable multilevel logistic regression, women age (20–35,36–49), wealth index (middle, rich), women number of children (have no chilled), household media exposure, husband age (21–30), women earn as compared to husband (less than, about the same), women contraception utilization (non-user), community level of illiteracy, countries (Mozambique and Tanzania) were significantly associated with women pressured to become pregnant by spouse and families “Table 4.”

**Table 4:** Multivariable multilevel logistic regression analysis of individual-level and community level factors associated with partner and family pressure to be pregnant among women in five Sub-Saharan Africa, DHS 2021–2023.

Individual level variables		Model I (AOR=95%)	Model II (AOR=95%)	Model III (AOR=95%)
Women age in years	15–19	1		
	20–35	1.5 (1.3,1.7)		**1.60 (1.46,1.87)** ^*^
	36–49	1.8 (1.5,2.1)		**1.93 (1.67,2.22)** ^*^
Wealth index	Poor	1		
	Middle	1.1 (1,1.2)		**1.12 (1.04,1.20)** ^*^
	Rich	1.1 (1,1.2)		**1.12 (1.03,1.21)** ^*^
Women education	No formal education	1		
	Primary	1.1 (1,1.1)		1.07 (0.99,1.15)
	Secondary	1 (0.9,1.1)		1.00 (0.90,1.10)
	Higher	0.9 (0.8,1.1)		0.90 (0.80,1.07)
Husband/Partner education	No formal education	1		
	Primary	0.9 (0.5,0.8)		1.02 (0.95,1.10)
	Secondary	0.9 (0.8,1.0)		0.96 (0.88,1.04)
	Higher	0.8 (0.7,0.9)		0.87 (0.76,0.98)
Women’s number of baby	Have no chilled	4.1 (3.7,4.6)		**4.65 (4.10,5.20)** ^*^
	Have Less than 5 baby	1.6 (1.5,1.7)		1.64 (1.50,1.77)
	Have More than or 5 baby	1		
Women media exposure	No	0.9 (0.8,0.9)		**.83 (0.78,0.88)** ^*^
	Yes	1		
Husband/partner age	Under 20	1		
	21-30	0.6 (0.5,0.8)		**0.74 (0.59,0.93)** ^*^
	31-39	0.7 (0.6,0.9)		0.89 (0.71,1.10)
	Above 40	0.8 (0.6,1)		1.02 (0.81,1.30)
Gender of house hold head	Male	0.9 (0.8,0.9)		0.96 (0.9,1.02)
	Female	1		
Partner/husband employment	Non-employed	1		
	Employed	1 (0.9,1.1)		1.07 (0.99,1.16)
Women earns as compared to husband/partner	Less than	0.7 (0.6,0.8)		0.78 (0.69,0.87)
	About the same	0.6 (0.5,0.7)		0.6 (0.53,0.67)
	More than	1		
Women utilization of contraception	User	1		
	Non-user	1.1 (1,1.2)		**1.06 (1.01,1.12)** ^*^
Community level variables				
Household residence	Urban		1.1 (1.1,1.2)	0.93 (0.81,1.03)
	Rural	1		
Community level of media exposure	Low		0.9 (0.8,1.1)	0.9 (0.96,1.3)
	High	1		
Community level of illiteracy	Low		1.5 (1.3,1.7)	**1.44 (1.25,1.66)** ^*^
	High	1		
Community level of poverty	Low		0.9 (0.8,1.1)	0.93 (0.83,1.05)
	High	1		
Country	Burkina Faso	1		
	Ghana		1.1 (1.1,1.2)	1.07 (0.99,1.17)
	Kenya		0.9 (0.8,1)	0.97 (0.87,1.07)
	Mozambique		1.7 (1.6,1.8)	**1.88 (1.7,2.03)** ^*^
	Tanzania		0.47 (0.4,0.5)	**0.45 (0.41,0.49)** ^*^

women to become pregnant was 1.6 and 1.93 times higher to pressurized among women aged 20-35 and 36-49 years compared to women aged 15–19 years (AOR = 1.6, 95% CI: 1.46,1.87) and 1.93, 95% CI:1.67,2.22), respectively. A women family living in middle and rich wealth index was both 1.12 times more likely to pressurized to become pregnant compared to women whose family living in a poor wealth index (AOR=1.12, 95% CI: 1.04,1.2) and (AOR:1.12, 95% CI: 1.03,1.21) respectively. Women pressured to become pregnant was 4.65 times more likely to occur among women who did not have children than among women who have children (AOR = 4.65, 95% CI: 4.1,5.2). The odds of women pressured to become pregnant were 17% less likely to occur among women who having media exposure compared to women not having media exposure (AOR = 0.83, 95% CI: 0.78,0.88). The odds of women pressured to become pregnant were 16% less likely to occur among women partner age 21–30 years compared to partner age of less than 21 or 31 years and above (AOR: 0.74, 95% CI: 0.59,0.93). Women pressured to become pregnant by husband and family was 1.06 times more likely to occur among women not using a contraceptive compared to user of a contraceptive method (AOR = 1.06, 95% CI: 1.01,1.12). women pressured to become pregnant by husband and family was 1.44 times higher to occur among women living in low level of community illiteracy compared to women living in high level of community illiteracy (AOR = 1.44, 95% CI:1.25,1.66). The odds of women pressured to become pregnant by husband and family were 1.88 times more likely to occur among women living in Mozambique and the odds of women pressured to become pregnant by husband and family were 55% less likely to occur compared to other Africa countries (AOR = 1.88, 95% CI:1.7,2.03) and (AOR= 0.45, 95% CI: 0.41,0.49), respectively “Table 4.”

## Discussion

The pressure for women to conceive, stemming from their partners and families, is a critical factor influencing maternal and fetal mortality rates in developing regions such as Sub-Saharan Africa. This study seeks to explore the occurrence of this pressure and the underlying contributing factors among married women in Sub-Saharan African countries. The research findings indicate that the prevalence of women facing pressure to become pregnant from their partners and families in Sub-Saharan Africa stands at 8.92% (95% CI: 8.74, 9.1). The observed rate of 8.92% for women facing pressure to become pregnant in Sub-Saharan Africa, as indicated in this study, contrasts significantly with findings from other regions. Previous studies in Nepal reported rates as high as 58% [44], while rates in five low and middle-income African countries ranged from 10.4% to 18% [45]. Similarly, a study in Northern California found a prevalence of 19% [46]. The lower prevalence in our study could be attributed to several factors. A key factor may be the utilization of a large sample size and the inclusion of women across all reproductive age groups. This differs from previous studies in certain African countries and Northern California, which had smaller sample sizes and limited age ranges among participants. By encompassing a wider demographic range, our study likely offered a more holistic insight into the issue, potentially reducing the pressure felt by women to conceive. The disparity between our study and the research conducted in Côte d’Ivoire and Kenya, where 6.4% to 7.8% [47], of women experienced pressure on their reproductive rights, suggests a higher prevalence in our findings. This variance could be due to their smaller sample sizes and exclusive focus on the need for contraception experience. Moreover, our study revealed a higher incidence of women coerced into pregnancy, with approximately 3% [48], of unintended pregnancies linked to partner pressure in Brazil city. The Brazil study’s narrow focus on pregnant mothers could potentially overlook non-pregnant women who faced pressure from partners or family members to conceive, leading to the discrepancy in results.

In the multivariable multilevel mixed-effect logistic regression analysis, factors such as women’s age, wealth index, number of children, media exposure, partner age, history of contraception utilization, community level illiteracy, and country category (specifically Mozambique and Tanzania) were found to be significantly associated with the pressure faced by reproductive age women in Sub-Saharan Africa countries to become pregnant by their spouses and families.

In this research, the probability of facing pressure to become pregnant was 1.6 times higher for women aged 20 to 35 and 1.93 times higher for those aged 36 to 49, in comparison to women aged 15 to 19. This aligns with previous studies [49–51]. The heightened pressure experienced by women in the 20–35 and 36–49 age brackets may be attributed to their perceived readiness for pregnancy, both psychologically and physically. Conversely, women who have not experienced pregnancy may encounter societal stigma and be labeled as infertile. This societal pressure may be particularly pronounced for married women beyond their teenage years, who might feel compelled to conceive shortly after marriage due to societal norms and limited decision-making autonomy. Such circumstances can increase the pressure to become pregnant and reduce the likelihood of contraceptive use

The probability of facing pressure to become pregnant was 1.12 times higher among reproductive-age women with middle and rich wealth status compared to those with poor wealth status. This trend may be linked to the financial stability of women with higher incomes, enabling them to potentially support a child more comfortably. As a result, partners and families may feel more inclined to pressure these women to conceive, overlooking their individual plans. Moreover, women with higher incomes are likely to afford contraceptive costs easily, as corroborated by other studies [52,53]. This indicates that women with greater financial resources are more susceptible to pressure from their partners and families to initiate pregnancy.

The likelihood of facing pressure to become pregnant was 4.65 times higher among reproductive-age women who had not yet given birth, compared to those who already had children. This assertion is reinforced by a study conducted in Ghana [54]. The pressure experienced by women without children may stem from perceived stress and concerns about infertility. There may be expectations from husbands to start a family and from family members to welcome grandchildren, contributing to heightened pressure on women who have not yet experienced childbirth to conceive.

The probability of experiencing pressure to become pregnant was 17% lower among women with media exposure compared to those without such exposure, consistent with prior research. This relationship may be attributed to women with media exposure being better informed about contraceptive methods and empowered to assert their reproductive rights, including accessing preconception care for improved birth preparedness. This empowerment can strengthen women’s decision-making skills [55,56]. Women who interact with media content may be more adept at handling pressure scenarios, enabling them to strategize and wait for the opportune moment for pregnancy. Furthermore, exposure to media can motivate women to proactively seek healthcare services before conceiving, receiving guidance on fertility from healthcare experts.

The probability of experiencing pressure to become pregnant was 26% lower among women whose husbands were aged between 21–30 years, in comparison to women whose husbands were older than 30 years. Women with husbands aged 21–30 years may benefit from better understanding and support from their partners, this supported by the study conducted in Iran [50,51]. These husbands may focus on employment to improve the family’s financial situation and may prioritize creating a stable foundation before starting a family. This could lead to a supportive environment where women are encouraged to use contraception and delay pregnancy, as they enjoy a period of happiness together before taking on the responsibilities of parenthood. On the other hand, as husbands’ ages increase beyond 30 years, there may be more pressure from them or their families for the women to conceive.

The likelihood of facing pressure to become pregnant was 1.06 times higher for women who were not using family planning methods or contraceptives compared to those who were using contraceptives [57–59]. This disparity may be attributed to the insufficient information about reproductive rights provided to women who do not use contraceptives by healthcare providers.

Women in communities with low levels of illiteracy are 1.44 times more likely to experience pressure to become pregnant compared to those in high-illiteracy communities. This disparity can be attributed to illiteracy, which significantly hinders women’s reproductive health and decision-making capabilities. Enhancing women’s literacy rates is crucial for empowering them to make informed choices regarding their reproductive health, access essential healthcare services, and advocate for their rights. This finding is supported by other research studies [59,60]. Educational and health literacy initiatives can play a vital role in helping women overcome these challenges and achieve improved reproductive health outcomes. Women in Sub-Saharan African countries residing in communities with low levels of illiteracy may have limited awareness of their reproductive rights.

The country of residence has been identified as a significant factor influencing the pressure faced by women of reproductive age to conceive in Sub-Saharan African nations. Women in Mozambique are 1.88 times more likely to experience pressure to become pregnant compared to their counterparts in Burkina Faso, Ghana, Kenya, and Tanzania. This difference may be linked to disparities in healthcare facility accessibility and lower socioeconomic status. Mozambique, potentially lacking in quality education, women’s empowerment initiatives, and reproductive health services, could contribute to this discrepancy [61,62]. On the other hand, women in Tanzania are 55% less likely to encounter pressure to conceive than those in Burkina Faso, Kenya, Mozambique, and Ghana. Tanzania’s strong educational system and societal development compared to other countries in the region may account for this variation [63,64].

## References

[1] TEAM UA. Delivering a world where every pregnancy is wanted every childbirth is safe and every young person’s potential is fulfilled.

[2] Arghavanian FE, Roudsari RL, Heydari A, Bahmani MND. Pregnant women’s experiences of social roles: an ethnophenomenological study. Iran J Nurs Midwifery Res. 2020;25(1):31–9. doi: 10.4103/ijnmr.IJNMR_54_19 31956595 PMC6952915

[3] Robinson GE. Stresses on women physicians: consequences and coping techniques. Wiley Online Library; 2003. p. 180–9. 10.1002/da.10069 12768652

[4] Barton K, Redshaw M, Quigley MA, Carson C. Unplanned pregnancy and subsequent psychological distress in partnered women: a cross-sectional study of the role of relationship quality and wider social support. BMC Pregnancy Childbirth. 2017;17(1): 1–9. doi: 10.1186/s12884-017-1223-x 28122585 PMC5267424

[5] Karaçam Z, Onel K, Gerçek E. Effects of unplanned pregnancy on maternal health in Turkey. Midwifery. 2011;27(2):288–93. doi: 10.1016/j.midw.2009.07.006 19773101

[6] Nelson JA, O’Brien M. Does an unplanned pregnancy have long-term implications for mother-child relationships? J Fam Issues. 2012;33(4):506–26. doi: 10.1177/0192513X11420820 28179747 PMC5293288

[7] Faisal-Cury A, Menezes PR, Quayle J, Matijasevich A. Unplanned pregnancy and risk of maternal depression: secondary data analysis from a prospective pregnancy cohort. Psychol Health Med. 2017;22(1):65–74. doi: 10.1080/13548506.2016.1153678 26920489

[8] Yanikkerem E, Ay S, Piro N. Planned and unplanned pregnancy: effects on health practice and depression during pregnancy. J Obstet Gynaecol Res. 2013;39(1):180–7. doi: 10.1111/j.1447-0756.2012.01958.x 22889435

[9] Spice K, Jones SL, Hadjistavropoulos HD, Kowalyk K, Stewart SH. Prenatal fear of childbirth and anxiety sensitivity. J Psychosom Obstet Gynaecol. 2009;30(3):168–74. doi: 10.1080/01674820902950538 19591052

[10] Taşdemir S, Balci E, Günay O. Comparison of life quality of pregnant adolescents with that of pregnant adults in Turkey. Ups J Med Sci. 2010;115(4):275–81. doi: 10.3109/03009731003628724 20828339 PMC2971486

[11] Tendais I, Figueiredo B, Mota J, Conde A. Physical activity, health-related quality of life and depression during pregnancy. Cad Saude Publica. 2011;27(2):219–28. doi: 10.1590/s0102-311x2011000200003 21359458

[12] Borcherding KE. Coping in healthy primigravidae pregnant women. J Obstet Gynecol Neonatal Nurs. 2009;38(4):453–62. doi: 10.1111/j.1552-6909.2009.01041.x 19614880

[13] Santelli J, Rochat R, Hatfield-Timajchy K, Gilbert BC, Curtis K, Cabral R, et al. The measurement and meaning of unintended pregnancy. Perspect Sex Reprod Health. 2003;35(2):94–101. doi: 10.1363/3509403 12729139

[14] Ameyaw EK, Budu E, Sambah F, Baatiema L, Appiah F, Seidu AA, et al. Prevalence and determinants of unintended pregnancy in sub-Saharan Africa: a multi-country analysis of demographic and health surveys. PLoS One. 2019;14(8):e0220970. doi: 10.1371/journal.pone.0220970 31398240 PMC6688809

[15] Fite RO, Mohammedamin A, Abebe TW. Unintended pregnancy and associated factors among pregnant women in Arsi Negele Woreda, West Arsi Zone, Ethiopia. BMC Res Notes. 2018;11(1):1–7. doi: 10.1186/s13104-018-3778-7 30223872 PMC6142678

[16] Omani-Samani R, Ranjbaran M, Mohammadi M, Esmailzadeh A, Sepidarkish M, Maroufizadeh S, et al. Impact of unintended pregnancy on maternal and neonatal outcomes. J Obstet Gynaecol India. 2019;69(2):136–41. doi: 10.1007/s13224-018-1125-5 30956467 PMC6430264

[17] Haddad LB, Nour NM. Unsafe abortion: unnecessary maternal mortality. Rev Obstet Gynecol. 2009;2(2):122–6. 19609407 PMC2709326

[18] Deller IG. Women’s experiences of being without children: William James College; 2017.

[19] Mackintosh M. Is there a ‘right’ time? Exploring women’s views and understandings on the timing of motherhood in Aotearoa, New Zealand; 2018.

[20] Gregson J. The culture of teenage mothers: State University of New York Press; 2010.

[21] Edac C, Andrade MLdS, Vitoriano LVT, Souza JdJ, Silva DOd, Gusmão MEN, et al. Association between unplanned pregnancy and the socioeconomic context of women in the area of family health. Acta Paulista De Enfermagem. 2012;25(S2):415–22.

[22] Deave T, Johnson D, Ingram J. Transition to parenthood: the needs of parents in pregnancy and early parenthood. BMC Pregnancy Childbirth. 2008;8:1–11. doi: 10.1186/1471-2393-8-30 18664251 PMC2519055

[23] Askew I, Maggwa N, Obare F. Fertility transitions in Ghana and Kenya: trends, determinants, and implications for policy and programs. Populat Development Rev. 2017;43(S1):289–307. doi: 10.1111/padr.12010

[24] Yeboah I, Kwankye SO, Frempong-Ainguah F. Predictors of underachieved and overachieved fertility among women with completed fertility in Ghana. PLoS One. 2021;16(6):e0250881. doi: 10.1371/journal.pone.0250881 34115779 PMC8195416

[25] Fincham FD, Beach SR. Conflict in marraige: implications for working with couples. Annu Rev Psychol. 1999;50(1):47–77. doi: 10.1146/annurev.psych.50.1.47 15012458

[26] Kluwer ES. From partnership to parenthood: a review of marital change across the transition to parenthood. J Family Theo Revie. 2010;2(2):105–25. doi: 10.1111/j.1756-2589.2010.00045.x

[27] NeJaime D. The nature of parenthood. The Yale Law J. 2017;2260–381.

[28] Amuyunzu-Nyamongo M, Biddlecom A, Ouedraogo C, Woog V. Qualitative evidence on adolescents’ views of sexual and reproductive health in Sub-Saharan Africa. 2005.

[29] Sodi T, Kpassagou LB, Hatta O, Ndayizigiye A, Ndayipfukamiye JM, Tenkué JN, et al. Parenting and parental burnout in Africa. New Dir Child Adolesc Dev. 2020;2020(174):101–17. doi: 10.1002/cad.20386 33206468

[30] Ward C, Makusha T, Bray R. Parenting, poverty and young people in South Africa: what are the connections? South African Child Gauge. 2015;1(1):69–74.

[31] Evans JL, Matola CE, Nyeko JP. Parenting challenges for the changing African family. Africa Future, Africa Challeng. 2008:265.

[32] Salami B, Alaazi DA, Okeke‐Ihejirika P, Yohani S, Vallianatos H, Tetreault B, et al. Parenting challenges of African immigrants in Alberta, Canada. Child Family Social Work. 2020;25(S1):126–34. doi: 10.1111/cfs.12725

[33] De Stone S, Meinck F, Sherr L, Cluver L, Doubt J, Orkin F, et al. Factors associated with good and harsh parenting of pre-adolescents and adolescents in Southern Africa. 2016.

[34] Goodkind D. The astonishing population averted by China’s birth restrictions: estimates, nightmares, and reprogrammed ambitions. Demography. 2017;54(4):1375–400. doi: 10.1007/s13524-017-0595-x 28762036

[35] Banerjee S, Alok S, George B. Determinants of women empowerment as measured by domestic decision-making: perspective from a developing economy. Advanced issues in the economics of emerging markets. Emerald Publishing Limited; 2020. p. 1–12.

[36] Leal Filho W, Azeiteiro U, Alves F, Pace P, Mifsud M, Brandli L, et al. Reinvigorating the sustainable development research agenda: the role of the sustainable development goals (SDG). Int J Sustainable Develop World Ecol. 2018;25(2):131–42. doi: 10.1080/13504509.2017.1342103

[37] Ragusa AT. Rurality’s influence on women’s intimate partner violence experiences and support needed for escape and healing in Australia. J Social Service Res. 2017;43(2):270–95. doi: 10.1080/01488376.2016.1248267

[38] Niolon P, Control CfD, Prevention. Preventing intimate partner violence across the lifespan: a technical package of programs, policies, and practices. Government Printing Office; 2017.

[39] Maharaj NR. Adolescent pregnancy in Sub-Saharan Africa–a cause for concern. Front Reproduct Health. 2022;4:984303. 10.3389/frph.2022.984303 PMC9755883 36531444

[40] Rutstein SO, Rojas G. Guide to DHS statistics. Calverton, MD: ORC Macro. 2006;38:78.

[41] Zegeye AF, Negash WD, Kassie AT, Wassie LA, Tamir TT. Home delivery among women who had optimal ANC follow-up in Sub-Saharan Africa: a multilevel analysis. PLoS One. 2023;18(11):e0295289. doi: 10.1371/journal.pone.0295289 38033152 PMC10688839

[42] Tesema GA, Mekonnen TH, Teshale AB. Individual and community-level determinants, and spatial distribution of institutional delivery in Ethiopia, 2016: spatial and multilevel analysis. PLoS One. 2020;15(11):e0242242. doi: 10.1371/journal.pone.0242242 33180845 PMC7660564

[43] Araújo MM, Botelho PB. Probiotics, prebiotics, and synbiotics in chronic constipation: Outstanding aspects to be considered for the current evidence. Front Nutr. 2022;9:935830. doi: 10.3389/fnut.2022.935830 36570175 PMC9773270

[44] Raifman S, Puri M, Arcara J, Diamond-Smith N. Is there an association between fertility and domestic violence in Nepal? AJOG Glob Rep. 2021;1(2):100011. doi: 10.1016/j.xagr.2021.100011 36276304 PMC9563493

[45] Stamatakis CE, Sumner SA, Massetti G, Kress H, Basile KC, Marcelin LH, et al. Sexual violence prevalence and related pregnancy among girls and young women: a multicountry analysis. J Interpers Violence. 2022;37(3–4):NP2428–41. doi: 10.1177/0886260520936366 32618217

[46] Miller E, Decker MR, McCauley HL, Tancredi DJ, Levenson RR, Waldman J, et al. Pregnancy coercion, intimate partner violence and unintended pregnancy. Contraception. 2010;81(4):316–22. doi: 10.1016/j.contraception.2009.12.004 20227548 PMC2896047

[47] Gelaw KA, Atalay YA, Gebeyehu NA. Unintended pregnancy and contraceptive use among women in low- and middle-income countries: systematic review and meta-analysis. Contracept Reprod Med. 2023;8(1):55. doi: 10.1186/s40834-023-00255-7 37993927 PMC10666441

[48] Pallitto CC, García-Moreno C, Jansen HAFM, Heise L, Ellsberg M, Watts C, et al. Intimate partner violence, abortion, and unintended pregnancy: results from the WHO multi-country study on women’s health and domestic violence. Int J Gynaecol Obstet. 2013;120(1):3–9. doi: 10.1016/j.ijgo.2012.07.003 22959631

[49] Mahapatro M, Gupta R, Gupta V. The risk factor of domestic violence in India. Indian J Community Med. 2012;37(3):153–7. doi: 10.4103/0970-0218.99912 23112440 PMC3483507

[50] Kargar Jahromi M, Jamali S, Rahmanian Koshkaki A, Javadpour S. Prevalence and risk factors of domestic violence against women by their husbands in Iran. Glob J Health Sci. 2016;8(5):175–83. doi: 10.5539/gjhs.v8n5p175 26652083 PMC4877196

[51] Ahmad J, Khan N, Mozumdar A. Spousal violence against women in India: a social–ecological analysis using data from the National Family Health Survey 2015 to 2016. J Interpersonal Violence. 2021;36(21–22):10147–81. 10.1177/0886260519881530 31642354

[52] Abate TW, Getahun B, Birhan MM, Aknaw GM, Belay SA, Demeke D, et al. The Urban–Rural differential in the association between household wealth index and anemia among women in reproductive age in Ethiopia, 2016. BMC Women’s Health. 2021;21:1–8. 34433446 10.1186/s12905-021-01461-8 PMC8386007

[53] Ewemooje OS, Biney E, Amoateng AY. Determinants of fertility intentions among women of reproductive age in South Africa: evidence from the 2016 demographic and health survey. J Pop Research. 2020;37(3):265–89. doi: 10.1007/s12546-020-09246-w

[54] Ahinkorah BO, Seidu AA, Armah-Ansah EK, Ameyaw EK, Budu E, Yaya S. Socio-economic and demographic factors associated with fertility preferences among women of reproductive age in Ghana: evidence from the 2014 demographic and health survey. Reprod Health. 2021;18(1):1–10. doi: 10.1186/s12978-020-01057-9 33388063 PMC7777390

[55] De Jesus M. The impact of mass media health communication on health decision-making and medical advice-seeking behavior of U.S. Hispanic population. Health Commun. 2013;28(5):525–9. doi: 10.1080/10410236.2012.701584 22888787

[56] Islam MM, Masud MS. Health care seeking behaviour during pregnancy, delivery and the postnatal period in Bangladesh: assessing the compliance with WHO recommendations. Midwifery. 2018;63:8–16. doi: 10.1016/j.midw.2018.04.021 29758443

[57] Kazmerski T, McCauley HL, Jones K, Borrero S, Silverman JG, Decker MR, et al. Use of reproductive and sexual health services among female family planning clinic clients exposed to partner violence and reproductive coercion. Matern Child Health J. 2015;19(7):1490–6. doi: 10.1007/s10995-014-1653-2 25416386 PMC10641793

[58] Silverman JG, Challa S, Boyce SC, Averbach S, Raj A. Associations of reproductive coercion and intimate partner violence with overt and covert family planning use among married adolescent girls in Niger. EClinicalMedicine. 2020;22:100359. doi: 10.1016/j.eclinm.2020.100359 32382722 PMC7198910

[59] Dadras O, Nakayama T, Kihara M, Ono-Kihara M, Dadras F. Intimate partner violence and unmet need for family planning in Afghan women: the implication for policy and practice. Reprod Health. 2022;19(1):52. doi: 10.1186/s12978-022-01362-5 35216612 PMC8881829

[60] Teshale AB. Factors associated with unmet need for family planning in Sub-Saharan Africa: a multilevel multinomial logistic regression analysis. PLoS One. 2022;17(2):e0263885. doi: 10.1371/journal.pone.0263885 35143584 PMC8830726

[61] Buur L, Salimo P. The political economy of social protection in Mozambique. 2018.

[62] Castel-Branco CN. Growth, capital accumulation and economic porosity in Mozambique: social losses, private gains. Rev African Polit Eco. 2014;41(sup1):S26–48. doi: 10.1080/03056244.2014.976363

[63] Wuyts M, Kilama B. The changing economy of Tanzania: patterns of accumulation and structural change: REPOA. 2014.

[64] Gupta V. A case study on economic development of Tanzania. J Int Acad Case Stud. 2020;26(1):1–16.

